# Spinal Nerve Root Haemangioblastoma Associated with Reactive Polycythemia

**DOI:** 10.1155/2014/798620

**Published:** 2014-11-06

**Authors:** Eric K. C. Law, Ryan K. L. Lee, James F. Griffith, Deyond Y. W. Siu, Ho Keung Ng

**Affiliations:** ^1^Department of Imaging & Interventional Radiology, Prince of Wales Hospital, The Chinese University of Hong Kong, 30-32 Ngan Shing Street, Shatin, New Territories, Hong Kong; ^2^Department of Radiology, Kwong Wah Hospital, Hong Kong; ^3^Department of Anatomical & Cellular Pathology, Prince of Wales Hospital, The Chinese University of Hong Kong, Hong Kong

## Abstract

Haemangioblastomas are uncommon tumours that usually occur in the cerebellum and, less commonly, in the intramedullary spinal cord. The extramedullary spinal canal is an uncommon location for these tumours. Also haemangioblastoma at this site is not known to be associated with polycythemia. We present the clinical, imaging, and histological findings of an adult patient with extramedullary spinal haemangioblastoma and reactive polycythemia. Radiography and computed tomography (CT) revealed a medium-sized tumour that most likely arose from an extramedullary spinal nerve root. This tumour appeared to be slow growing as evidenced by the accompanying well-defined bony resorption with a sclerotic rim and mild neural foraminal widening. Magnetic resonance imaging revealed prominent flow voids consistent with tumoural hypervascularity. CT-guided biopsy was performed. Although preoperative angiographic embolisation was technically successful, excessive intraoperative tumour bleeding necessitated tumour debulking rather than complete tumour resection. Histology of the resected specimen revealed haemangioblastoma. Seven months postoperatively, the patients back pain and polycythemia have resolved.

## 1. Introduction

Haemangioblastoma is a benign hypervascular neoplasm that can occur anywhere in the neuroaxis, most commonly in the posterior cranial fossa and within the spinal cord [1]. Up to one-third of haemangioblastomas are associated with von Hippel-Lindau disease [1, 2]. Of those arising in the spinal cord, three-quarters arise in an intramedullary location while one-quarter arises in an extramedullary location which includes the cauda equina, filum terminale, and rarely the proximal ends of the exiting nerve roots [2]. As far as we are aware, no previous case of extramedullary spinal haemangioblastoma associated with polycythemia has been reported. We herein present such a case.

## 2. Case Summary

A 59-year-old man with type II diabetes mellitus and hypertension was admitted for nontraumatic low back pain of one month's duration. The pain was progressive in nature and associated with numbness and weakness of the right foot. There were no bowel or urinary symptoms. He was a nonsmoker and nondrinker and worked as a construction site worker. There was no significant family history. Physical examination revealed decreased pin-prick sensation in the L4 and L5 dermatomes. The rest of the neurological examination was normal.

Full blood count revealed polycythemia with a haemoglobin of 23.8 g/dL (normal upper haemoglobin limit is 17.2 g/dL). All other laboratory investigations, including the 24-hour urine catecholamine level, were normal. Bone marrow examination revealed no evidence of a myeloproliferative malignancy, indicating that this was a reactive polycythemia.

**Figure 1:**
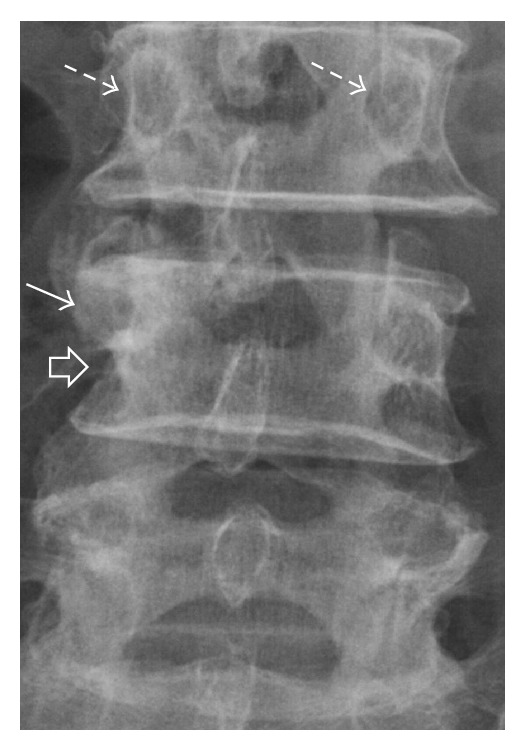
Localised anteroposterior view of lumbosacral radiograph showing erosion of the lateral half of the right L4 pedicle (white arrow), lateral border of the vertebral body (open arrow), and associated bony sclerosis. Note the normal, symmetrical pedicles at L3 (dashed arrows).

**Figure 2:**
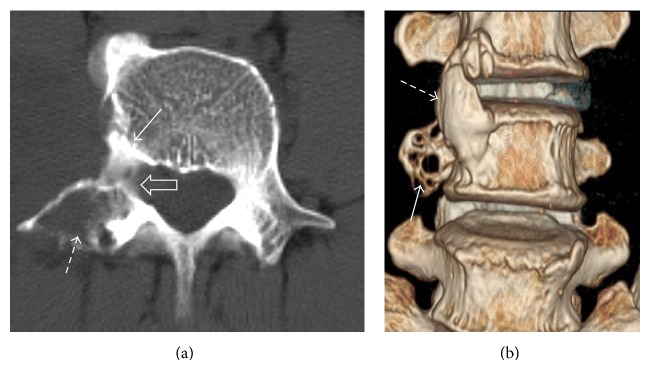
Axial CT image (a) at L4 shows bone erosion (dashed arrow), sclerosis (white arrow), and widening of the exit foramina (open arrow). Volume rendered three-dimensional coronal image (b) demonstrates the focal erosion at the transverse process (white arrow) and anterolateral osseous bridging between the L3 and L4 vertebral bodies (dashed arrow).

**Figure 3:**
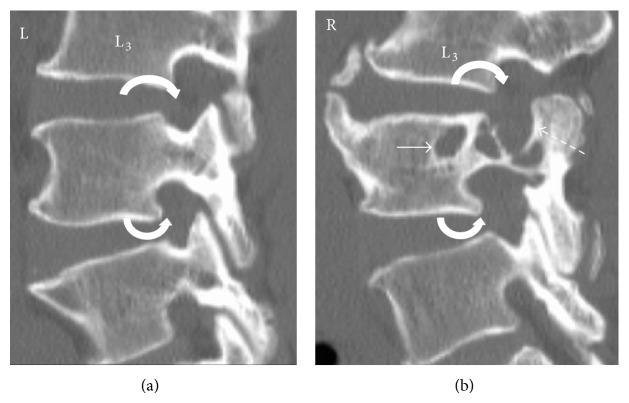
Coned-in sagittal-oblique reformatted CT images of L3 to L5 (bone window) showing the normal left and affected right side with pressure erosion of the L4 vertebral body (white straight arrow) and its posterior elements (dashed arrow). Moderate widening of the right L3/4 and L4/5 exit foramina (curved arrows) is present compared to the normal left side.

**Figure 4:**
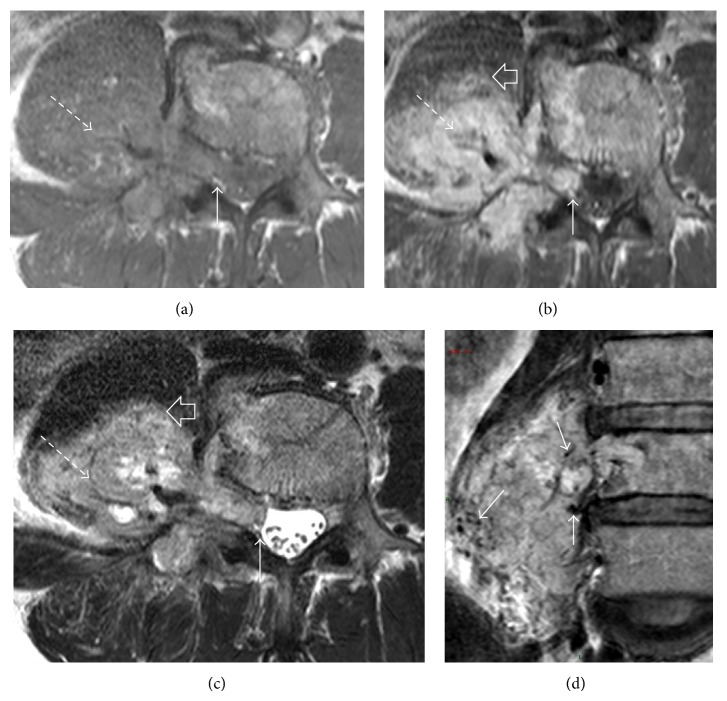
MRI at L4 lumbar level with axial T1W (a), T1W after contrast (b), and T2W (c) shows a T1-hypointense, T2-heterogenous paraspinal tumour with avid enhancement (dashed arrow). It extends into the moderately widened neural foramen with central displacement of the thecal sac (arrow). The tumour infiltrates the psoas muscle (open arrow). Coronal T2-weighted image (d) demonstrates hypointense tubular structures within the tumour (arrows) compatible with flow voids, indicative of hypervascularity.

Lumbosacral spine radiograph showed erosion of the right L4 pedicle and the right lateral vertebral body wall of L4 (Figure 1). Computed tomography (CT) revealed well-defined osteolysis on the right side of the vertebral body and posterior elements of L4 with a well-defined sclerotic border (Figures 2 and 3) as well as moderate widening of the L3/4 and L4/5 exit foramina and osseous bridging on the anterolateral aspect of the L3 and L4 vertebral bodies. On MRI, there was a medium-sized (5.8 cm wide × 4.7 cm deep × 7.4 cm long) T1W isointense, T2W heterogeneous extraforaminal paravertebral tumour at the L4 level associated with internal tubular flow voids and avid contrast enhancement (Figure 4). The tumour infiltrated the ipsilateral psoas muscle, L4 posterior elements, and vertebral body with further extension to the right L3/4 and L4/5 lateral recess and exit foramina, which were moderately widened. There was encasement of the exiting right L4 and descending right L5 nerve roots. The overall appearances were consistent with a slow growing, nonaggressive tumour such as a high-flow vascular malformation or a paraganglioma. Although a hypervascular metastatic deposit such as from a renal cell carcinoma was considered, the longstanding nature of the imaging features was against this diagnosis. CT-guided biopsy revealed a highly vascularised tumour, consisting of sheets of plump polygonal to spindle cells supported in a richly vascularised stroma. Immunohistochemistry staining raised the possibility of metastatic renal cell carcinoma or paraganglioma. PET-CT (figure not shown) revealed mild increase in 18-F FDG uptake with a SUV 2.5 of the L4 paraspinal tumour. No other hypermetabolic focus was present. There were no cystic lesions in the pancreas or the kidneys to suggest underlying von Hippel-Lindau disease. CT brain (not shown) excluded a cerebellar haemangioblastoma.

**Figure 5:**
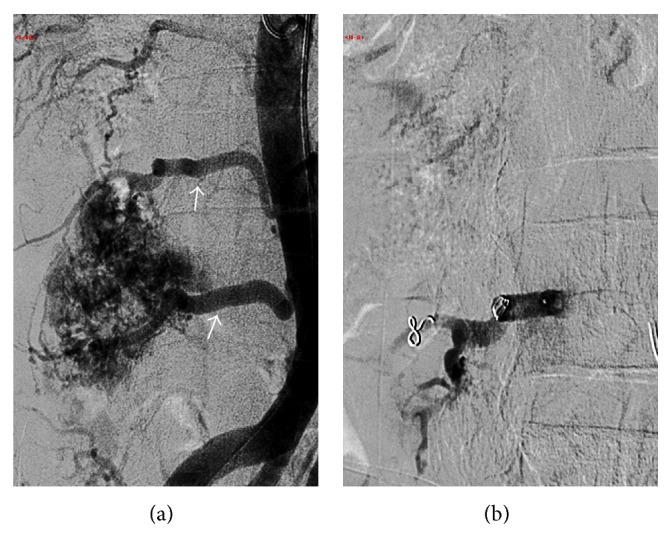
Frontal angiographic images preembolization (a) shows two large L3 and L4 lumbar arteries (white arrows) supplying the hypervascular tumour. Postembolisation with particles (150–250 microns) and fiber coils (b) shows near complete obliteration of the tumor vascularity.

**Figure 6:**
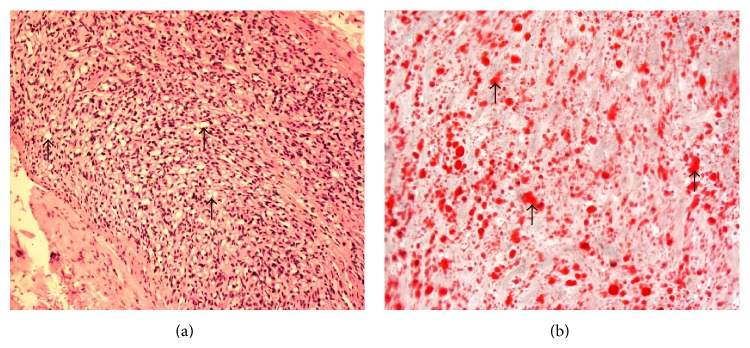
100x magnification of the H & E section (a) showing numerous fine capillaries (dark purple dots) separated by stromal cells with vacuolated cytoplasm (nonstained areas, black arrows). Oil Red O stain (×400 magnification; (b)) demonstrates tumour cells that are lipid laden (all regions stained red, illustrated with arrows). Overall features are compatible with a haemangioblastoma.

Preoperative angiography revealed that the hypervascular tumour was supplied by moderately hypertrophied L3 and L4 lumbar arteries. Particle and coil embolisation of these feeder arteries was performed 16 hours prior to surgical resection (Figure 5). Surgery was technically difficult as the tumour was closely related to the exiting lumbar nerve roots. This, together with heavy blood loss (3L) from the tumour bed, led to only subtotal tumour excision being performed. Histologically, the resected specimen comprised sheets of tumour cells with clear cytoplasm separated by a rich network of thin capillaries, with intervening fibrovascular and mature fatty tissue (Figure 6). Immunohistochemistry demonstrated positive staining for CD34 (a marker of early hematopoietic tissue) and negative urogenital (cytokeratins and PAX8), muscle (SMA, desmin, and myogenin), and melanocytic markers (HMB45, melanA, and MITF). The overall features were compatible with a haemangioblastoma.

**Figure 7:**
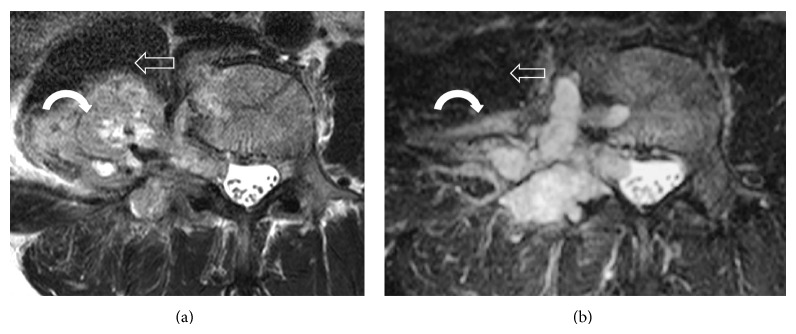
Comparison of the preoperative (a) and postoperative (b) T2W MR image at the L4 vertebral body level, demonstrating a reduction in tumour size (curved arrow) after partial tumour resection. In particular, the invasion into the psoas muscle (open arrow) is less.

Back pain, numbness, and weakness were significantly improved on clinical follow-up seven months after surgery while the polycythemia had completely resolved. The haemoglobin level also normalised one month after surgery (12.6 g/dL) and remained normal on further follow-up (seven months after operation), demonstrating how the polycythemia was secondary to the spinal haemangioblastoma. Follow-up MRI lumbar spine six months after operation showed a marked reduction in the size of the right paraspinal tumour (Figure 7). The current management plan is to repeat the MRI examination and full blood count in one year to monitor tumour size and haemoglobin level.

## 3. Discussion

Spinal haemangioblastoma occurs less frequently than its intracranial counterpart and comprises about 1.6–5.8% of all spinal cord tumours [1–4]. Within the spine, most (75%) haemangioblastomas are intramedullary in location and are usually located in the dorsal aspect of the cervical and thoracic segments of the spinal cord. The remainder of spinal haemangioblastomas are extramedullary in location arising from the cauda equina, filum terminale, and, rarely, the proximal ends of the exiting nerve roots [2, 5, 6]. A mixed location pattern can also exist with a single tumour having both intramedullary and extramedullary components. There are fewer than fifty cases of exiting nerve root haemangioblastomas [4–7] reported and none of these, to our knowledge, has been associated with reactive polycythemia. Herein, we presented the first case of an extramedullary spinal haemangioblastoma with reactive polycythemia. Two features helped confirm that the reactive polycythemia was secondary to this tumour, namely, normalisation of polycythemia postoperatively and positive CD34 tumour staining, which is a marker of early haematopoietic cell line activity. While 10–20% of von Hippel-Lindau associated cerebellar haemangioblastomas are associated with reactive polycythemia due to an erythropoietin (or an erythropoietin-like substance) released from the tumour [3, 7], this is the first reported case of extramedullary spinal haemangioblastoma associated with reactive polycythemia.

Histologically, the surgical specimen demonstrated histologic features similar to those found in cerebellar and intramedullary spinal cord haemangioblastomas with characteristic clear stromal cells admixed with a rich capillary network [8, 9]. Although percutaneous biopsy tissue yield was reasonable, definitive preoperative histological diagnosis was not possible due largely to the relatively small amount of tissue on which to base a definitive histological diagnosis. This is similar to the experience of others [10, 11], who also found that a definitive histological diagnosis usually relied on the surgically resected specimen.

Haemangioblastomas have a propensity for the central nervous system with immunohistochemical studies demonstrating markers of vascular, glial, neural, fibrohistiocytic, and smooth muscle cell lineages [8, 9]. Their occurrence in atypical locations can potentially harbor both diagnostic and therapeutic pitfalls. In a literature review of 85 spinal haemangioblastomas, Browne et al. [4] found that only 7% lesions were truly extramedullary in position and that these extramedullary haemangioblastomas were solid in nature as opposed to intramedullary spinal and intracerebral haemangioblastomas, which are typically mainly cystic tumours [4–7]. Although we did not make an imaging preoperative diagnosis in this case, there were two particular imaging features present which would seem helpful in making this diagnosis if similar tumours are encountered in future. First the well-defined osteolysis with a sclerotic rim as well as neural foraminal widening strongly indicates a tumour of longstanding nature, helping to exclude other hypervascular tumours such as metastatic renal cell or thyroid carcinoma. Second, flow voids are an uncommon feature of soft tissue tumours in general and are not recognized features of other tumours occurring in a paraspinal location such as schwannoma or neurofibroma. The main differential diagnosis on imaging is spinal paraganglioma which can also be associated with tumoural flow voids, intense enhancement, and pressure erosion of the adjacent vertebrae [10]. The normal 24-hour urine catecholamine level and the lack of chief cells in the final histology help to exclude this diagnosis [10, 11]. The flow voids also prompted us to undertake preoperative angiography and embolisation, which is a useful adjunct to surgical resection of hypervascular tumours [12]. In a small case series of four extramedullary haemangioblastomas (one in the conus medullaris, one in the cauda equina, and two in the filum terminale) embolisation was successful in three patients (75%) facilitating complete tumour removal [12]. Similar results of successful embolization were also observed in three intramedullary haemangioblastomas and one combined intra/extramedullary tumour [13]. Despite a technically successful embolisation 16 hours prior to surgery in our patient, significant intraoperative blood loss (3L) was still encountered such that only partial tumour excision could be achieved. As the patient is now largely symptom-free, there is no plan for further surgical intervention. Although this tumour was hypervascular, its indolent preoperative imaging features would tend to suggest that tumour regrowth following debulking surgery is likely to be slow, though there are no reports of partially resected extramedullary tumours to provide guidance in this respect.

## 4. Conclusion

We present a rare case of extramedullary haemangioblastoma associated with reactive polycythemia. Preoperative imaging indicated features which should prompt consideration of this tumor when similar cases are encountered in future. The clinical symptoms and polycythemia resolved with tumor debulking surgery.
